# Minimally invasive computer-assisted repair of feline sacroiliac luxation—a cadaveric study

**DOI:** 10.3389/fvets.2025.1528345

**Published:** 2025-02-27

**Authors:** Nicole Diana Wolf, Lukas Kleiner, Christina Precht, Julien Guevar, Mathieu de Preux, Franck Forterre, Pia Duever

**Affiliations:** ^1^Division of Small Animal Surgery, Department of Clinical Veterinary Medicine, Vetsuisse-Faculty, University of Bern, Bern, Switzerland; ^2^Divison of Small Animal Surgery, Department of Clinical Veterinary Medicine, Tierklinik Marigin, Feusisberg, Switzerland; ^3^Divison of Clinical Radiology, Department of Clinical Veterinary Medicine, Vetsuisse-Faculty, University of Bern, Bern, Switzerland; ^4^Divison of Small Animal Neurology, Department of Clinical Veterinary Medicine, Anicura Tierklinik Thun, Thun, Switzerland; ^5^Swiss Institute of Equine Medicine (ISME), Department of Clinical Veterinary Medicine, Vetsuisse-Faculty, University of Bern, Bern, Switzerland

**Keywords:** minimally invasive, computer-assisted orthopedic surgery (CAOS), sacroiliac luxation, feline, Stealth Station S8, cadaveric study

## Abstract

**Introduction:**

The delicate anatomy of the feline sacrum presents challenges for surgeons to perform a safe and accurate surgery without risking to damage vital neurovascular structures. In this context computer-assisted surgery represents an attractive minimally invasive surgical solution to increase the accuracy and safety of the intervention. This cadaveric study evaluates the feasibility and safety of a minimally invasive approach by a novice surgeon using computer navigation compared to traditional fluoroscopy as well as a new method for patient reference array positioning.

**Material and methods:**

Eleven cats' cadavers were used to simulate sacroiliac joint luxation whereas one had to be excluded due to a sacral fracture. Sides were randomly assigned to two groups: (1) minimally invasive computer-assisted drilling group (MICA group); (2) fluoroscopy-controlled group (FC group). All surgeries were performed by a first-year ECVS resident. After positioning of the reference array, cone beam computer tomography scans were conducted for planning of the temporary and final fixation of the sacroiliac luxation. Final fixation was achieved through a minimally invasive approach via computer-assisted drilling of the iliac wing and the sacral body for the placement of a positional screw (2.4 mm). The other side was operated on via an open dorsal, fluoroscopy-controlled approach. Comparison between the two groups for surgical time, accuracy of screw placement, radiologic safety and the learning curve was recorded. Statistical analysis consisted of Fisher's exact test to compare the assigned radiological safety grades and the Wilcoxon signed-rank test for total surgery time and accuracy.

**Results:**

Mean total time for MICA and FC groups were 44 min and 45 s and 19 min and 54 s, respectively. The mean total time for the first five cases was 53 min and 30 s in the MICA group and 20 min and 15 s in the FC group and improved to a mean total time of 36 min and 15 s in the MICA group and to 18 min and 40 s in the FC group in the second five cadavers. Accuracy aberration of surgery in the MICA group improved from a mean deviation on the target point, the end of the drill tract, from 4.2 mm in the first five to 0.9 mm in the second five cats. This criterion was only applicable in the MICA group. Evaluation for radiologic safety was assessed with three radiologic categories (I-III) and four subcategories (a-d). Additionally, the surgery was classified into radiographically safe implant placement (yes/no). The first five cats of the MICA as well as the FC group received a lower safety grade compared to the second five cats. The novel method for placement of the patient reference array was categorized as grade I without violating any vital structures in all 10 cats.

**Discussion:**

The computer-assisted surgery for minimal invasive surgical fixation of sacroiliac luxation seems to be a safe procedure with a steep learning curve. Compared to previous study using the same technical set-up, the safety of the computer-assisted surgical procedure was improved by changing the smooth to the negative threaded pin to have better bone purchase for sufficient anchoring in the spinous process alone and therefore minimizing the risk for violation of the spinal canal.

## 1 Introduction

Sacroiliac luxation is a common injury in cats, especially after high velocity trauma like road traffic accident or high-rise trauma. Sacroiliac luxation occurred in 59.2% of cats with pelvic injuries and accounted for 27% of all pelvic fractures. Sacroiliac separation was the second most common injury after pelvic floor fractures (1, 2). Treatment options are either conservative or surgical. If displacement is minimal or the luxation is unilateral with no concurrent pelvic fractures it might be treated conservatively with analgesics, cage rest and monitoring of urination and defecation (3). Surgical stabilization of sacroiliac luxation is indicated if there are signs of severe pain, inability to ambulate, neurologic deficits attributable to the luxation, severe instability or displacement (>50%) of one or both hemipelvises, pelvic outlet obstruction and/or concurrent orthopedic injuries (1, 4, 5).

The method of choice for surgical fixation is a cortical screw in lag fashion, or alternatively in a positional fashion (1, 2). Furthermore, a single transiliosacral pin in cats or a transiliosacral rod in dogs as well as transiliosacral toggle sutures, transileac pin/bolt/screw and tension band technique are described for successful stabilization (6–10). The fixation can be achieved through an open, dorsal or ventral, or closed, minimally invasive, approach with or without the help of fluoroscopy (3, 4, 11–16).

Since the sacral body of cats is very small, penetrating vital structures like the spinal canal dorsally, the intervertebral disc cranially and important nervous and vascular structures ventrally while placing the lag screw into the sacral body is a feared complication. Therefore, accurate positioning is essential, best at the first attempt (17, 18).

The correct position of the screw can be secured either through fluoroscopic control or as shown by the study of Kleiner et al. with computer-assisted surgery using an optical tracking system, the StealthStation (12, 16, 19, 20). However, this study showed, that placement of the patient tracker pin could increase the risk of complications by penetrating the spinal canal to achieve enough stability of the patient tracker. Open reduction techniques are invasive and correct screw placement can be challenging.

Previous studies showed that closed reduction and stabilization provided more accurate and consistent screw placement along safe corridors and optimal sacral purchase with minimal tissue dissection (12, 16, 20–24). Further documented benefits in human surgery are reduced blood loss, shorter surgical and hospitalization times, improved pain scores, faster weight bearing, lower complication rates and lower costs (25–32).

To the authors knowledge there is no study demonstrating the feasibility and safetiness of a minimal invasive approach for the fixation of the sacroiliac joint by computer-assisted drilling with any surgical navigation system. The hypothesis of this study is that minimal invasive computer-assisted surgery is more accurate than fluoroscopy-controlled drilling for the repair of sacroiliac joint luxation and that the procedure might be conducted by an unexperienced surgeon.

## 2 Material and methods

### 2.1 Preparation of cadavers

The study considered cadavers of 11 skeletally mature cats with an intact pelvic region that died or were euthanized for reasons unrelated to this study. Exclusion criteria were all pelvic or sacral pathologies like pelvic fractures, sacroiliac luxation, lumbosacral (sub) luxation and sacral fractures. Therefore, all cadavers underwent examination with a cone beam computed tomography (CBCT) scan beforehand. One cat had to be excluded due to an accidentally induced sacral fracture while manually luxating the sacroiliac joint during preparation which resulted in 10 cats included in the study.

The cat cadavers were donated to our institution by the owners who gave consent to use them for research purposes.

All images (Fluoroscopic and CBCT) were generated remotely without exposing personnel to radiation.

The first side was operated with the new computer-assisted technique through a minimal invasive approach (minimal invasive computer-assisted surgery, MICA). The second side of each cat was operated using an open dorsal, fluoroscopy controlled, approach (fluoroscopy-controlled surgery, FC). As a result, a total of 20 surgeries were carried out. The sides with which to start with (right or left sacroiliac joint) were randomized.

All joints of all cats were luxated manually with a Freer periosteal elevator through a ventral abdominal approach as described by Borer et al. (3, 11) and Montavon et al. (39).

For comparable stability during the surgeries, the first sacroiliac joint was luxated and surgically stabilized with the positional screw before the second side was luxated and fixated. Using a ventral abdominal approach, the pelvic symphysis was separated by an oscillating saw to imitate additional pelvic fractures (Colibri II; DePuy Synthes, West Chester, PA). Following this, only one side was luxated during each surgery.

### 2.2 Technical equipment, orthopedic equipment, implants

**Figure 1:**
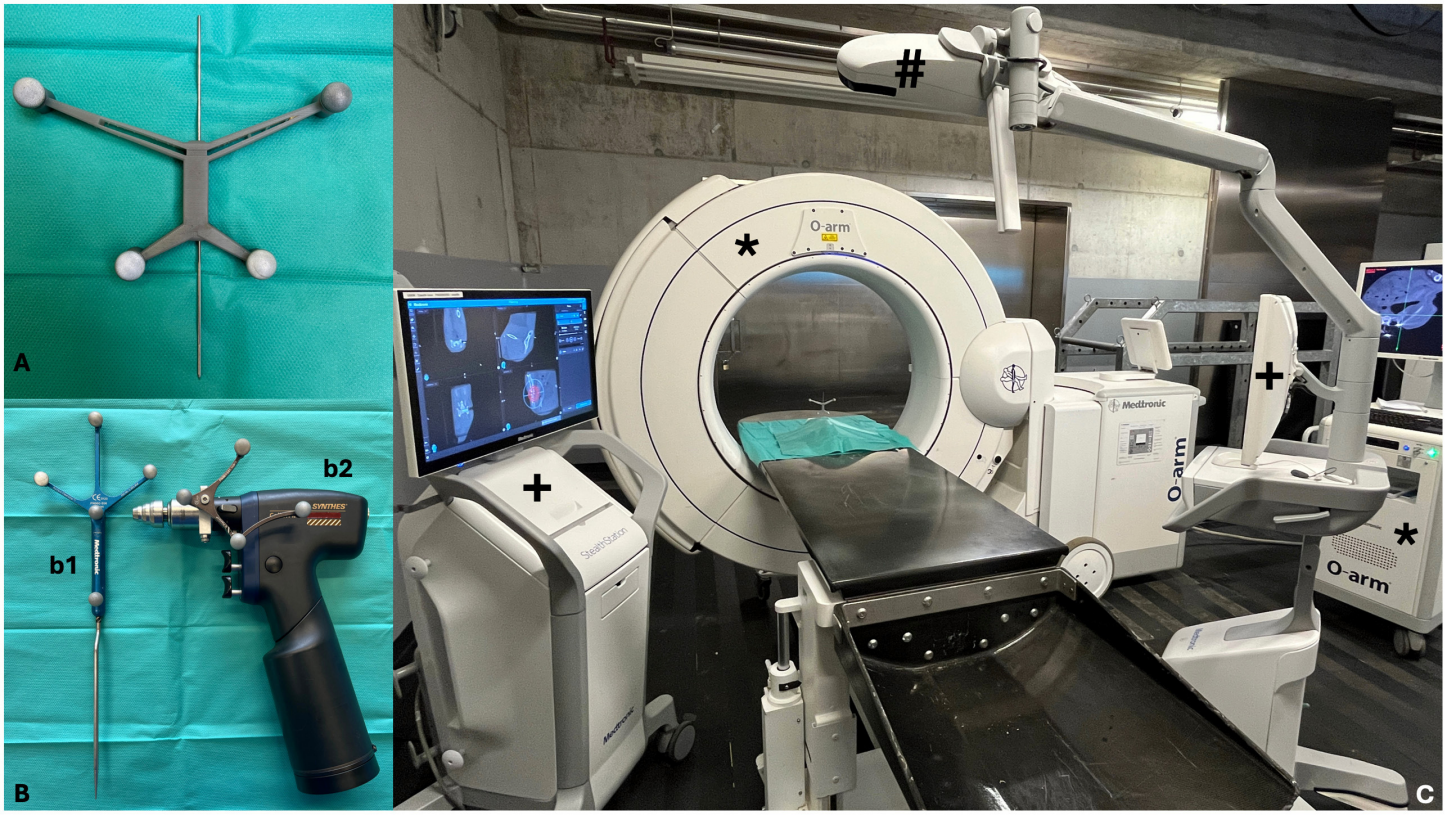
**(A)** 3D printed patient reference array with its reflecting spheres and pre-positioned 1.4 mm negative threaded pin. **(B)** Specific instrumentation for the navigated procedure: (b1) Navigated Pointer (Passive Planar Marker); (b2) Instrument tracker on a battery-powered drill (SureTrakII clamps and tracker on Colibri II). **(C)** Set-up for the computer-navigated drilling: O-arm with screen (*), Stealth Station S8 with its two screens (+), infrared camera for optical tracking (#).

Images for the minimally invasive computer-assisted surgical repair of sacroiliac luxation were acquired with a mobile cone beam computed tomography unit (CBCT; O-Arm; Medtronic, Louisville, Colorado), which was coupled to a surgical navigation system equipped with an optical tracking system, the StealthStation S8 (Medtronic). Specific instrumentation for the navigated procedure included a patient reference array, a navigated pointer (Passive Planar Marker, Medtronic) and an instrument tracker (SureTrak II clamps and tracker, Medtronic) mounted on a battery-powered surgical drill (Colibri II, DePuy Synthes, West Chester, PA) (Figure 1). Every instrument and tracker that had to be detected by the localizer camera of the optical tracking system was mounted with infrared reflecting spheres, also called fiducials. Furthermore, a radiolucent carbon fiber table (Opera Swing; General Medicale Merate SPA, Seriate, Italy) was used.

A lighter, custom-made copy of the original patient reference array was 3D printed to avoid excessive lever arm on the small feline sacral bone. The patient reference array was a modification of the version used by Papacella-Beugger et al. (33), printed out of Formlabs Tough 1500 and weighted 9 g. The array was equipped with six holes through the middle segment to press fit a 1.4 mm negative threaded stainless-steel pin for the fixation into the sacral spinous process.

Further surgical instruments included a Backhaus clamp and a kern bone holding forceps, a 1-0 Kirschner (K)-wire, tap sleeves of the according size, 1.1 and 1.8 mm drill bit, a 2.4 mm self-tapping cortical screw, a screwdriver and a pin cutter.

### 2.3 Surgical procedure

All surgeries were performed by a first year European College of Veterinary Surgery (ECVS) resident without significant experience in orthopedic surgical procedures. For both procedures (i.e., the computer-assisted and the fluoroscopic guided drilling) the cats were placed in lateral recumbency on the carbon fiber table whereas the correct positioning with superimposition of the transverse processus of the seventh lumbar vertebra was evaluated through fluoroscopy.

#### 2.3.1 Image acquisition, surgical planning, patient registration, and instrument calibration

One latero-lateral fluoroscopic projection was performed to confirm the correct positioning of the sacroiliac region of the cadaveric specimen in the isocenter of the gantry of the O-arm. A navigated 3D CBCT-scan (high resolution scan of 192 images during one tube rotation with an exposure of 120 kV and 20 mA and a voxel size of 0.4 × 0.4 × 0.8 mm^3^) was then acquired. During the scan acquisition, the camera of the StealthStation S8 had to detect simultaneously the tracker of the O-arm gantry and the patient reference array. The acquired CBCT data set was automatically transferred to the navigation system.

Preoperative surgical planning of the drill corridor was performed using the planning function of the Stealth Station S8. The entry point was set on the lateral aspect of the ilial wing whereas the target point marked the end of the drill hole at 50% of the depth in the sacral body. The planning of the drill corridor was done on the axial and coronal view and verified with probe's eye view.

**Figure 2:**
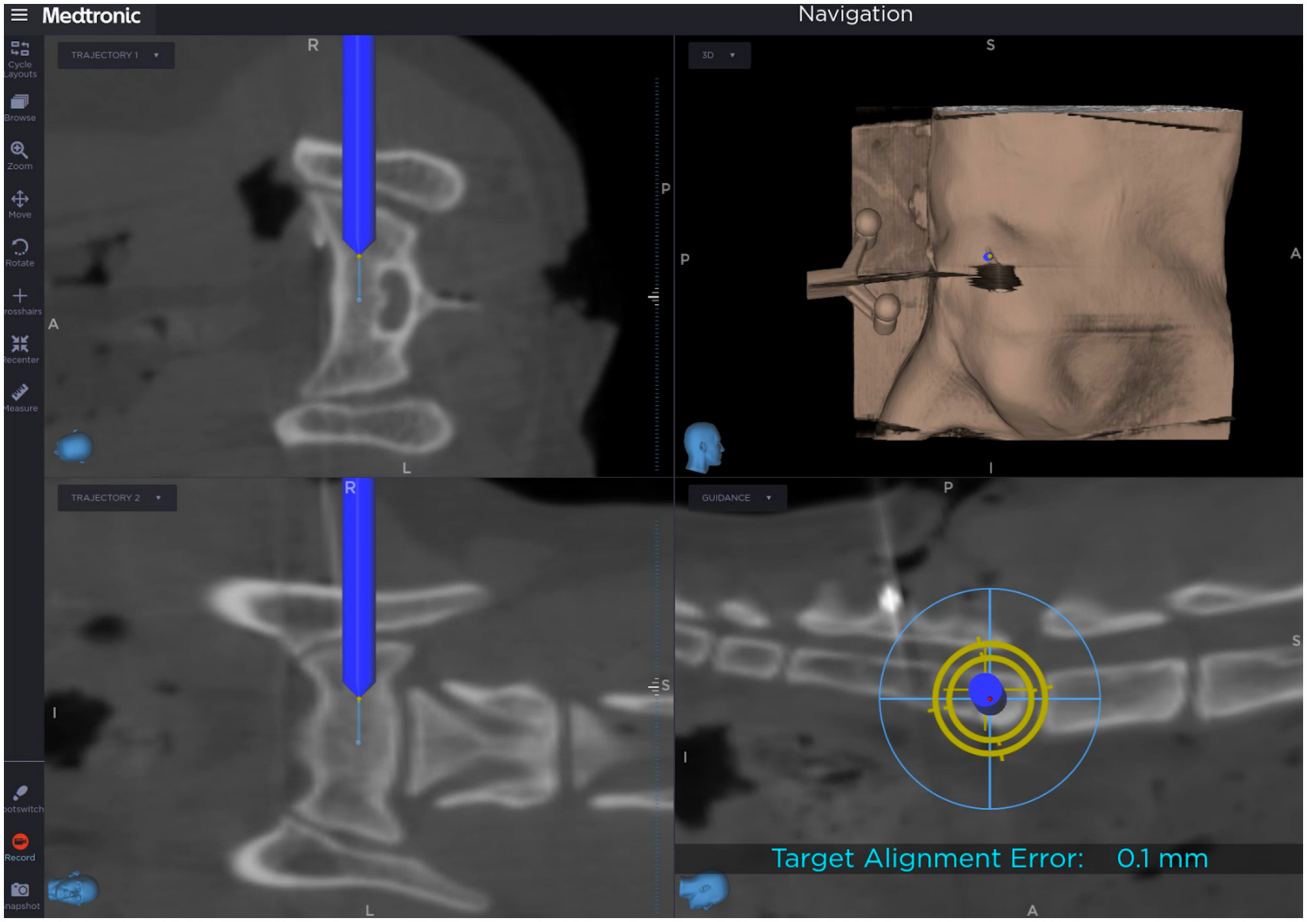
Screen view during computer-assisted drilling in trajectory 1 and 2 as well as guidance view. The dark blue cylinder represents the drill bit following the planned drill corridor (light blue line).

The O-arm was then moved away from the surgical area to provide unrestricted access to the surgical site. To start the navigated surgical procedure, patient registration (32) was performed by touching the divot of the patient reference array with the tip of the navigated pointer. Instrument calibration was performed by following consecutive steps instructed by the StealthStation S8 for the identification of the plane, tip and long axis of the instrument. Calibration was repeated whenever a drill bit of different length was used, in order to display the correct length on the screen of the navigation system. For intraoperative, real-time orientation, the “navigation” mode was selected, and the trajectory 1 and 2 as well as the guidance function were displayed on the screen (Figure 2).

#### 2.3.2 Minimally invasive computer-assisted drilling

The location of the spinous process of the first and second sacral vertebra was determined by percutaneous palpation. Decision to use S1 or S2 was made individually depending on the size of S1 or S2 on each cadaver, whereas the larger spinous process was selected for patient reference array placement.

A stab incision to the cortex of the spinous process was performed using a #11 scalpel blade. A negative threaded 1.4 mm pin with the pre-positioned reference array and its fiducials was drilled at a 45-degree angle from ipsilateral through the spinous process and, in contrast to the study of Kleiner et al., not anchored into the roof of the sacrum (19). The reference array was moved away from the surgical site by bending the anchoring pin to prevent spatial interference with the navigated instruments and ensure continuous detection by the localizer camera throughout the procedure.

The first CBCT scan was conducted to plan the location in the caudal part of sacrum for the temporary fixation pin to hold the reduction in place. Reduction of the sacroiliac luxation was achieved with the help of a Backhaus clamp in the ischial tuberosity to facilitate movement of the bone while manually pushing the wing of the ilium in its desired place. Assessment of reduction was estimated with the symmetrical palpation of the iliac wings in its cranial and dorsal plane.

The navigated pointer was used to determine the location of the entry point according to the predefined plan on the StealthStation 8. A stab incision was then performed with a #11 scalpel blade onto the near cortex of the ilium in the correct location as seen on the screen for temporary fixation pin placement. A 1.1 mm tap sleeve was placed and the location and direction of the pin placement was verified again with the help of the navigated pointer and the 1-0 K-wire was drilled through the tap sleeve in the desired direction to temporarily keep the ilium to the sacrum.

Another CBCT scan was made to assess the reduction and create the plan for the computer-assisted drilling of a hole through the ilium and sacrum for correct screw placement in the center of the sacral body, as described by Burger et al. (17). If the reduction was not accurate, the previously described procedure had to be repeated.

After planning and calibration of the instruments, the hole in the ilium and sacrum was drilled under surgical navigation guidance. Stability of the patient reference array and thus accuracy of the navigation system was verified before each drilling by touching the entry point of the patient reference array pin with the navigated pointer and by simultaneously assessing the virtual images displayed on the screen of the navigation system.

The first three cats were directly drilled with a 1.8 mm drill bit of 100 mm in length. As slippage of the drill bit on the iliac wing as well as bending of the drill was evident, the following seven cats were predrilled with a smaller 1.1 mm drill bit of 37 mm in length and the use of a 2.0 mm tap sleeve to protect the soft tissues. The first hole was enlarged with a 1.8 mm drill bit. A 2.4 mm self-tapping positional cortical screw of appropriate length was placed.

The conducted drill holes aimed to reach only 50% of the width of the sacral body to not interfere with the subsequent contralateral procedure.

#### 2.3.3 Fluoroscopy controlled drilling

A conventional open dorsal approach to the sacroiliac joint was chosen. To allow good visualization of the sacral wing the iliac wing was retracted caudoventrally by an assistant. A surgical power drill (Colibri II) was used to place a 0.8 mm K-wire in the sacral wing to facilitate the correct screw placement through the sacral body according to Burger et al. (17) via fluoroscopic control. The K-wire placement was targeted at the center of the first sacral body. Fluoroscopic control was performed after every new pin positioning. Once the correct positioning of the pin was achieved, the pin was removed and the hole within the sacral body was sequentially overdrilled with a 1.1 mm and a 1.8 mm drill bit. Reduction of the luxation was carried out under visualization and held in place by the assistant until the equivalent hole in the wing of the ilium was drilled with a 1.8 mm drill bit and the 2.4 mm self-tapping positional cortical screw of appropriate length was placed.

### 2.4 Time

For all procedures “total time” was noted and split into “time on patient” and “time off patient”. “Total time” started with the placement of the patient reference array (MICA group) or the manipulation of the previously luxated sacroiliac joint (FC group). It ended in both groups after the placement of the 2.4 mm positional screw. “Time on patient” was defined as time needed for the surgical procedures which includes surgical approach, placement of the reference array, reduction and temporary fixation of the luxation, the drilling process itself and placement of the screw. “Time off patient” was defined as time needed for the imaging procedures in both groups (CBCT scans and fluoroscopy) and the planning of temporary fixation and the drill hole in the MICA group.

The additional control CBCT scans taken only for radiologic evaluation were not included in any of the time measurements.

### 2.5 Accuracy

**Figure 3:**
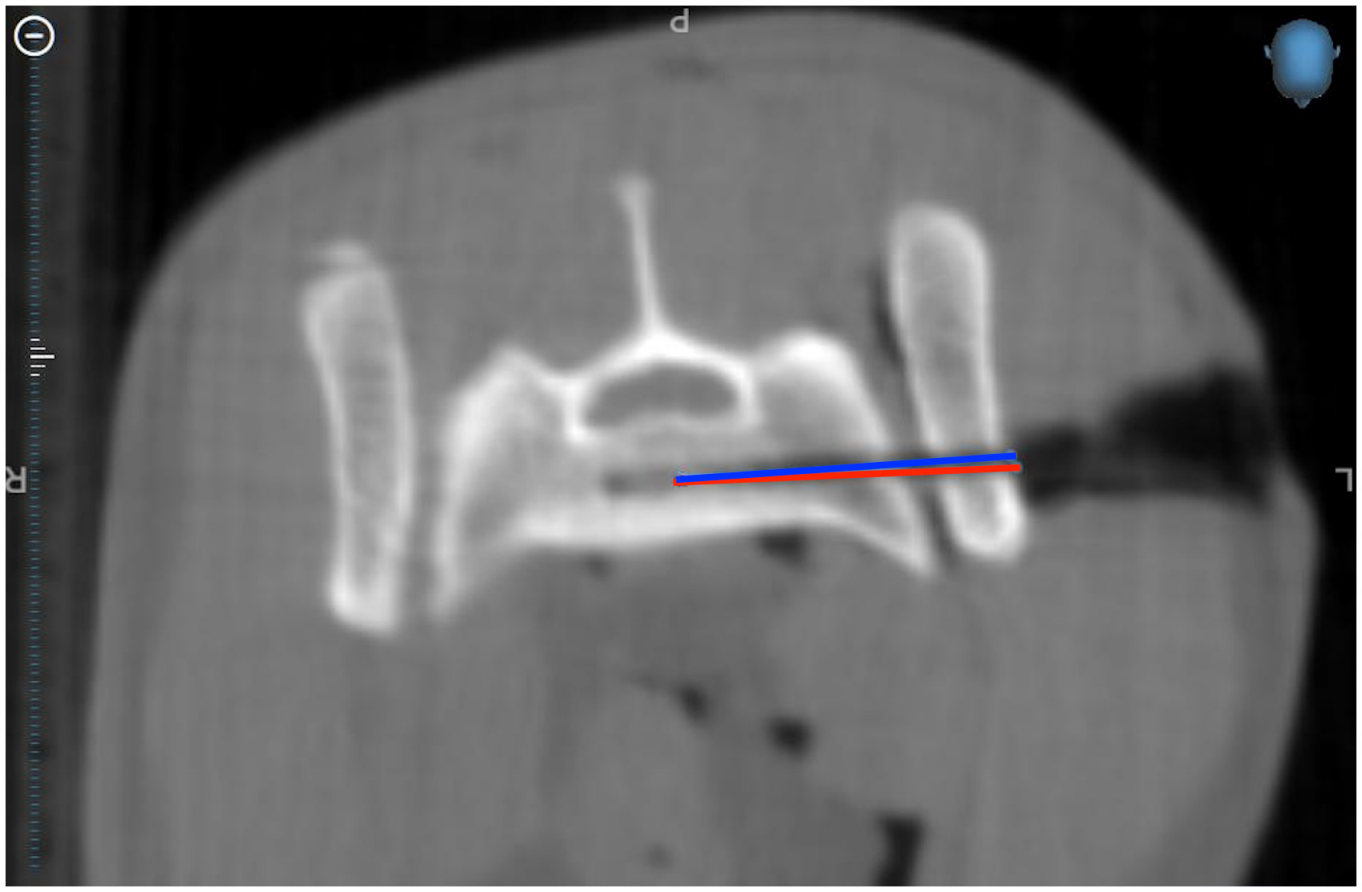
Merged CBCT images where you can see the planned drill corridor (red) and the actual drill corridor (blue) in Cat 10.

The accuracy of the drilling was evaluated for the MICA group where the initial plan and the actual drill hole could be compared via CBCT images (Figure 3). Entry and target points for the conducted drill hole were defined on postoperative CBCT images using Stealth Navigation software (Medtronic). To compare the initial plan and the actual drill hole, pre- and postoperative CBCT images had to be manually merged to achieve superimposition of the images so that 3D coordinates could be extracted. To calculate the deviation in millimeters between the initial plan and the actual drill hole, the coordinates of the entry and target points were described by the Euclidean distance formula


$$\begin{array}{l} \sqrt{{\left(X2-X1\right)}^{2}+{\left(Y2-Y1\right)}^{2}+{\left(Z2-Z1\right)}^{2}} \end{array}$$

to calculate the deviation in millimeters to compare the initial plan and the actual drill hole (33, 34).

### 2.6 Radiologic evaluation

To ensure an optimal radiologic evaluation, five additional CBCT scans were conducted after drilling the hole in the sacral body. These included: one scan after completing the computer-assisted drill hole, one scan with the screw placed on the first side, one scan following the fluoroscopy guided freehand drill hole with the first screw still in place, one scan with the first and second screw in place, and the final scan without any implants, showing only the two drill holes.

The postoperative CBCT images were evaluated by a Diplomate of the European College of Veterinary Diagnostic Imaging (DECVDI), C.P. The primary focus was the course of the drill hole in the 20 surgical procedures and the positioning of the patient reference array in the 10 cats in the MICA-group. Radiologic safety was assessed using three categories (I–III) and four directional subcategories (a–d) (Table 1). Additionally, surgeries were classified based on radiographic safety of implant placement (yes/no). Category I represented a drill hole entirely within the vertebral body, category II was assigned if the screw was in contact or eroding the sacral cortex, and category III indicated a violation of the cortex. Categories I and II were considered radiographically safe, whereas category III was classified as radiographically unsafe. The subcategory described the direction of deviation: a = dorsally, b = ventrally, c = cranially, d = caudally. As the drill holes only penetrated 50% of the sacral body to avoid contralateral interference, radiologic evaluation extended to 60% of bone purchase, as recommended for fixation of sacroiliac luxation.

**Table 1:** Safety grade drilling corridor.

**Safety grade**	**Safety name**	**Description**
I	Well IN (optimal placement)	Trajectory within the vertebra, intact cortex of the vertebral canal floor and the ventral aspect of the sacrum
IIa	Just, IN	Trajectory within the vertebra, in contact or eroding the cortex of the vertebral canal floor
IIIa	Too far, IN	Trajectory violating the cortex of the vertebral canal floor or within the vertebral canal
IIb	Just, OUT	Trajectory within the vertebra, in contact or eroding the ventral aspect of the sacrum
IIIb	Too far, OUT	Trajectory violating the ventral cortex of the sacrum or ventrally outside of the sacrum
IIc	Just, CRAN	Trajectory within the vertebra, in contact or eroding the cranial endplate of S1
IIIc	Too far, CRAN	Trajectory violating the cranial endplate of SI or within the IVDS L7/S1
IId	Just, CAUD	Trajectory within the vertebra, in contact or eroding the caudal endplate of S1
IIId	Too far, CAUD	Trajectory violating the caudal endplate of SI or within S2

The safety of the patient reference array was categorized into grade I, IIIa, or IIIb. Grade I was assigned if the patient reference array was located within the dorsal spinous process of S1 or S2. Grade III indicated sacral cortex penetration, with IIIa specifying intrusion into the vertebral canal and IIIb indicating direction toward the soft tissues ventral to the sacrum.

### 2.7 Statistical analysis

The learning curve was analyzed by comparing the first five to the last five surgeries. Wilcoxon signed-rank test was used to compare the two groups concerning the total surgery time. More insight was gained by dividing the total time between the time spent on the cadaver and the time spent planning the surgery. The same method was used to compare the deviation from the target for the screw entry and target sites. Fisher's exact test was performed to compare the frequency of the assigned radiological safety grades. The analysis was performed using the statistical software R (version 4.3.1) in Rstudio (Posit Software, version 2023.09.1.494).

## 3 Results

The cadavers of 10 mature domestic shorthair cats were included in the study, seven males and three females with a median weight of 3.88 kg (range 2.1–6.3 kg).

Comparison of the first five procedures to the second five showed an improvement in total time, accuracy and safety, especially in the MICA group.

Inadequate reduction of the sacroiliac luxation was detected on preprocedural CBCT scan and had to be corrected in two cases (Cat 1 and Cat 3). Reduction was adequate in the remaining 8 cats.

### 3.1 Time

The MICA group showed a significantly slower “total time” (*p* = 0.002) with a mean of 44 min 45 s ± 11 min 53 s compared to the mean “total time” of 19 min 54 s ± 6 min 58 s in the FC group.

For the MICA group the mean “time on patient” was 23 min 50 s ± 8 min 55 s and the “time off patient” was 20 min 40 s ± 5 min 43 s. For the FC group, both, time on-and off-patient were shorter, with the “time on patient” being 14 min 40 s ± 4 min 42 s and “time off patient” of 4 min 40 s ± 3 min 40 s.

In the MICA group, although not statistically significant (*p* = 0.056), the mean “total time” decreased from 53 min 30 s ± 8 min 20 s to 36 min 15 s ± 8 min 40 s in the first vs. the second five procedures. A less pronounced improvement was seen in the FC group from the first five cases with a mean “total time” of 20 min 30 s ± 3 min 45 s to 18 min 50 s ± 9 min 20 s in the second five procedures, this was also not statistically significant (*p* = 0.31).

When comparing “time on patient” between the first five and the second five procedures, neither the MICA group (*p* = 0.222) nor the FC group (*p* = 0.69) resulted in a statistically significant difference. The “time on patient” decreased in the MICA group from 28 min 20 s ± 10 min 30 s to 19 min 36 s ± 6 min 10 s whereas the FC group showed a minimal improvement in “time on patient” from 15 min 15 s ± 4 min to 14 min 16 s ± 5 min 43 s.

The reduction from the mean “time off-patient” of 24 min 55 s ± 3 min 40 s in the first five procedures to a mean “time off-patient” of 16 min 30 s ± 3 min 35 s in the second five procedures in the MICA group was statistically significant (*p* = 0.032). This comparison in the FC group did not show a significant improvement (*p* = 0.222) with a mean “time off-patient” of 5 min 10 s ± 7 min 42 s in the first five compared to the “time off-patient” of 4 min 34 s ± 4 min 40 s in the second five cases.

### 3.2 Accuracy

This criterion was only measurable in the MICA group.

The 10 cases in the MICA group showed a mean deviation of 2 mm at the entry point and a mean deviation of 2.5 mm at the target point. Although not statistically significant (*p* = 0.063), there was an improvement in the accuracy aberration when comparing the first five to the second five procedures.

The mean deviation on the entry point decreased from 2.4 to 1.6 mm and on the target point from 4.2 to 0.9 mm in the first five compared to the second five procedures.

### 3.3 Radiologic evaluation

In all cats, the safety grade for placement of the patient reference array was categorized as I, with a trajectory running correctly within the dorsal spinous process of S1 or S2.

Inferior safety grade for the screw placement was assigned to the first three cadavers of both groups with a IIIb in the MICA and IIb in the FC group compared to grade I in the remaining seven cadavers.

**Figure 4:**
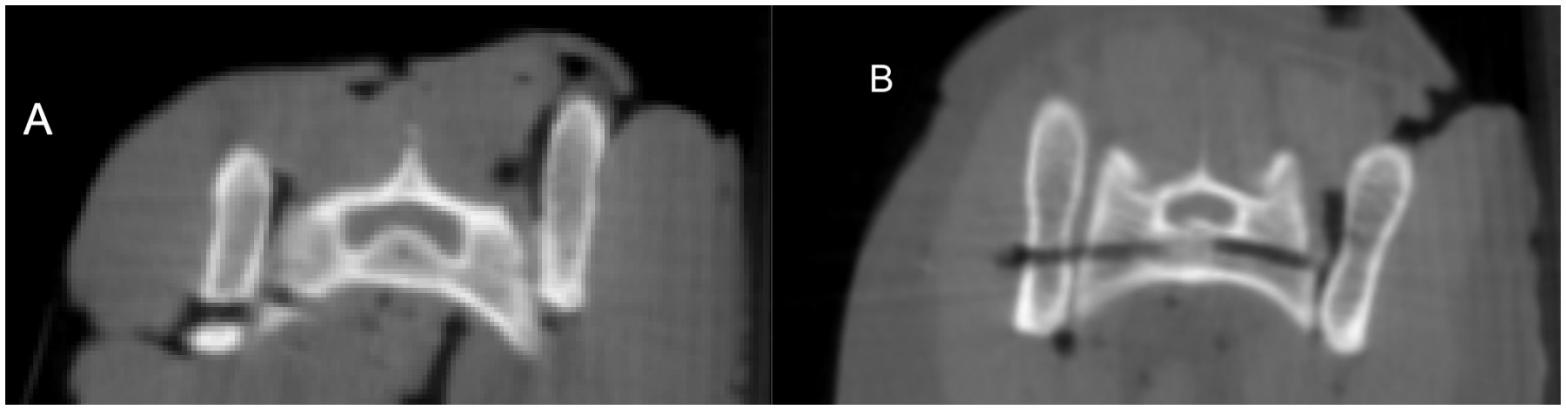
Final CBCT scans for radiologic evaluation without implants. **(A)** Drill corridor graded as IIIb where it ended ventrally outside of the sacrum and was categorized as radiographically unsafe (Cat 2, MICA procedure). **(B)** Drill corridor graded as I where it runs within the vertebrae and was categorized as radiographically safe (Cat 9, MICA procedure on the left side of the image and FC procedure on the right side of the image).

The safety grade in the MICA group was worse in the first five trials compared to the last five ones. Three cadavers (3/10) were categorized a IIIb, whereas the trajectory was violating the ventral cortex of the sacrum or was even ventrally outside of the sacrum and therefore rated as radiographically unsafe implant placement. The following seven cadavers (7/10) were classified in category I and fulfilled the criterion for radiographically safe implant placement (Figures 4A, B).

In the FC group the first and third cat were categorized as IIb with the trajectory being in contact or eroding the ventral cortex of the sacrum (2/10). All the other were evaluated as category I (8/10), which means the procedures on all 10 cadavers achieved radiographically safe implant placement.

The radiologic grading wasn't statistically significant when performing the Fisher's exact test due to a small sample size (count range 2–7 for the different grades).

## 4 Discussion

Computer-assisted surgery for minimally invasive surgical fixation of feline sacroiliac luxation seems to be a safe procedure with a steep learning curve for a novice surgeon. Several cadaver surgeries were needed to get comfortable with the handling of the technical instruments and the navigation software. In our experimental set-up, the reduction and surgical fixation of sacroiliac luxation via an open dorsal, fluoroscopy-controlled approach also resulted in safe implant placement in most of the cadaveric specimens. Although the minimally invasive procedure was associated with a significantly longer surgical time, it avoids the creation of a large surgical approach and improves intraoperative control of implant placement.

Compared to the study of Kleiner et al. (19) the safety of the computer-assisted surgery was improved by changing the smooth to the negative threaded pin to have better bone purchase for sufficient anchoring in the spinous process alone and therefore minimizing the risk of violation of the spinal canal.

The technical equipment used from Medtronic is very user-friendly. The only downside was the recognition of the custom-made 3D printed patient reference array by the Stealth Station Navigation system in some trials. In one case (Cat 6) it took 15 min and several adjustments in position of the infrared camera for the Stealth Station to recognize the patient reference array and to conduct a computer navigated scan. In other trials, one of the four reflecting spheres had to be removed for the infrared camera to recognize the patient reference array. These challenges did prolong the “time off patient” but did not interfere with the accuracy. As previously described, the patient reference array was a modification to the version used by Papacella-Beugger et al. (33) Our patient reference array was out of Formlabs Tough 1500 and weighted 9 g whereas the one from Papacella-Beugger is out of polyactide with a weight of 12 g compared to the original patient reference array by Medtronic with 59 g. Modification included the fixation with a press fit negative-threaded pin going through the patient reference array compared to a pre-printed fixation method with variable adapters by Papacella-Beugger. Challenges in recognition of the custom-made patient reference array by the Stealth Station Navigation system were not described in their study and we assume it to be due to a slight deviation in the geometry of the arms of the patient reference array resulting from 3D printing.

The positioning of the patient reference array is a critical point in computer-assisted surgery. An accurate correlation between the CBCT images and therefore the plan and the anatomical structures in the surgical field are only given, if the patient reference array is securely anchored to the target bone throughout the whole procedure, in an angle-stable manner. If any alteration of the spatial relationship between the array and the target bone occurs, the CBCT scans must be redone and a new plan according to the new reference has to be made. In the study of Kleiner et al. the reference array was fixated with a smooth pin which was drilled through the spinous process of S1 or S2 and anchored in the roof of the sacrum (19). As in some of the first cases the pin penetrated the spinal canal, the fixation of the reference array was changed for our study. We used a negative threaded pin to improve stability and eliminating the need for a second anchoring point into the roof of the sacrum due to better bone purchase of the threaded version. As a result, positioning in the spinous process was sufficiently stable and the safety of the procedure was increased. None of the pins in the present study violated any important structures. Furthermore, by placing the patient reference array in the same bony structure as the target bone (Spinous process of S1 or S2 for surgeries on the sacral body), a potential error in surgical accuracy aberration was minimized. As described in other studies (35, 36), applying pressure on the bone during surgical manipulation can alter its imaged position if the patient reference array is fixed to a different structure than the target structure. If the patient reference array is fixed to the same structure where the surgery takes places, movement of it correlates with the image due to a similar movement of the reference array. Nevertheless, movement and manipulation of the imaged position should be minimized.

As demonstrated in previous studies, a minimally invasive approach for the repair of sacroiliac joint luxation led to more accurate screw placement and less soft tissue damage and therefore a reduction of patient morbidity might be anticipated (12, 16, 20, 23, 24). The control for screw placement was usually done by fluoroscopy. The safety of screw placement in sacroiliac luxation with the help of computer navigation was proven in a study by Kleiner et al. (19), which lead to a combination of a minimally invasive approach with the guidance of computer navigation. The basics of the minimal invasive computer-assisted surgical procedure are similar to the minimal invasive approach described by Tomlinson et al. (12). The advantage of this new technique is the control of reduction via CBCT before fixation and the precise planning of the drill corridor through the ilium and sacral body while sparing the soft tissue. However, for cases with a longer duration from trauma to surgical repair, the surgeon should be prepared to convert to an open approach if a closed approach is not amenable for adequate reduction screw placement.

Due to the CBCT scans, planning process and set up of the technical equipment of the surgical navigation, the total time is doubled in the MICA group compared to the FC group, but with training a clear reduction in time is anticipated as seen when comparing the first five to the second five trials. Thus, training and practice are essential to optimize time when using computer navigation. Similar results were seen in the study of Kleiner et al. (19) with a mean duration of 23 min and 37 s for the computer navigated group and 9 min and 47 s in the FC group with improvement of time in both groups with training. However, a team approach should be considered when applying this technique in clinical cases to optimize the workflow of the navigated procedure in order to shorten the overall surgery time and minimize the associated complications (37).

The observed surgical accuracy aberration from the planned to the actual drilled corridor of 2 mm at the entry point and 2.5 mm at the target point were similar to the results with an open approach computer-assisted surgery shown by Kleiner et al. with a deviation of 2 mm at the entry point and 1.6 mm at the target point in the same anatomical location (19). Comparable results were achieved using the same CBCT-based computer-assisted drilling procedure in other anatomical locations (33, 34). Papacella-Beugger had a median entry point deviation of 1.8 mm (range: 0.3–3.7 mm) and median exit point deviation of 1.6 mm (range: 0.6–5 mm) in lumbar plate fixation (33). In the study by Guevar et al. for minimally invasive stabilization in the thoracolumbar spine the overall mean deviations for the entry points were 2.2 mm for the experienced surgeon and 3.7 mm for the novice surgeon. For the exit points, deviations were 3.0 and 5.0 mm, respectively. Significant difference was found in accuracy between the experienced and novice surgeon for the deviations overall (34). An accuracy aberration of 2 and 2.5 mm in entry and target point matches the accuracy expectations when applying an optical tracking system (like the Stealth Station) for a computer-assisted orthopedic surgical procedure (38). Overall, a deviation in the close range of 2 mm meets the high standards in precision required when working on a bony structure as small as a feline sacrum. Comparison of drill hole accuracy between an experienced surgeon vs. a lesser experienced surgeon in this anatomical location was performed by Kleiner et al. and did not show a significant difference (19). Therefore, this study was carried out by novice first year ECVS resident surgeon only.

The first three cadavers with the highest deviation on entry as well as target point and the poorest radiology safety grade (IIIb in MICA Group/IIb in FC Group) were drilled with a 1.8 mm drill bit. As slippage of the drill bit on the iliac wing was evident, the following 7 cats where predrilled with a smaller 1.1 mm drill bit and showed less deviation from the initial plan and scored a better radiological safety grade. The 1.1 mm drill bit was shorter than the 1.8 mm drill bit (35 vs. 100 mm). It was found that longer drill bits tend to bend more easily and therefore the actual position of the drill tip does not match with the fiducials on the handpiece and the drilling is not accurate to the planned procedure on the screen. Similar observations with bending of the drill were made in equine computer-assisted surgeries, whereas recommendation to use a shorter drill bit was expressed (35). Nevertheless, the lack of surgical experience in drilling uneven, slippery surface on the iliac wing as well as first using a longer drill bit is hypothesized to have influenced the results more than the lack of experience with the navigation system as demonstrated by improvement of accuracy with the transition to a shorter drill bit and more training.

To facilitate radiologic evaluation, account for possible artifacts due to the metal implants and avoid interference between the bilateral drill holes, additional CBCT scans were performed after the surgical procedures with the implants in place and after removal. In a clinical setting, only one CBCT scan would typically be required for temporary fixation without computer navigation, or two scans if computer navigation is used, as opposed to the seven CBCT scans performed in this study. Since time measurement was paused for the additional CBCT scans, total time could only be reduced by omitting the scan for the temporary fixation pin planning. Since there are no critical structures located directly ventral to the vertebra, a previous study by Shales et al. (18, 21) did not consider screws penetrating the ventral cortex as mispositioned. Nevertheless, we did categorize our first three cadavers as a radiographically unsafe implant placement because there was not enough bone purchase before violating the ventral cortex and stability of the fixation could not be ensured as well as neurologic deficits could not be excluded in this *ex vivo* study. We did consider Category I and II as radiographically safe implant placement where the cortex was not violated, and a good surgical outcome would be expected. Due to possible neurological deficits and screw loosening and therefore unsatisfactory surgical outcome, category III was classified as radiographically unsafe implant placement. Depending on the direction of deviation (a = dorsally, b = ventrally, c = cranially, d = caudally) in category III a revision would be needed or even a fatal outcome could be anticipated.

### 4.1 Limitations

One of the main limitations of the study is its cadaveric nature with artificially induced unilateral sacroiliac luxation which does not provide an accurate imitation of a clinical case as muscle trauma and additional pelvic fractures are not identical. The feasibility has to be tested for the closed reduction of the sacroiliac luxation in an *in vivo* study where muscle contracture is present or if a bilateral luxation, and therefore change in known landmarks, complicates the surgery. Furthermore, both sides of each cat were used for this study, this could cause a bias in anatomical landmarks when doing the second procedure. Cautious extrapolation of the results of this experimental study to clinical cases is warranted, and the potential for screw loosening and neurological deficits should be assessed in an *in vivo* study, particularly in cats that were categorized in group IIIb where the trajectory violated the ventral cortex.

Different surgical approaches (minimally invasive vs. open approach) were used for the two different groups and therefore limited an exact comparison concerning time and visualization of anatomical structures. As all surgeries were performed by a first-year surgery resident without major orthopedic experience, some initial problems were evident, like slippage of the drill bit on the bony surface. On the other hand, a steep learning curve was observed. Results could differ if a more experienced surgeon had performed the surgeries.

To allow direct comparison of the plan and the actual drillhole the pre- and postoperative CT images were manually merged using Stealth Station software and could therefore lead to an error in accuracy.

The effect of increased exposure to radiation of patients treated by MICA in comparison to those of the FC group should also be taken in consideration but was not estimated in the present study. This has yet to be tested.

## 5 Conclusion

Computer-assisted surgery for minimally invasive sacroiliac luxation in cats can be considered as a safe alternative procedure with a high accuracy for correct screw positioning. The time needed for surgery is increased compared to fluoroscopy controlled freehand surgery but a steep learning curve positively affecting accuracy and duration of the procedure was observed, leading to the assumption that with more experience the observed results might be further improved and further emphasize the advantages of not performing a large open approach, especially regarding intra- and postoperative morbidity.

## Data Availability

The original contributions presented in the study are included in the article/supplementary material, further inquiries can be directed to the corresponding author.
